# A framework for advancing our understanding of cancer-associated fibroblasts

**DOI:** 10.1038/s41568-019-0238-1

**Published:** 2020-01-24

**Authors:** Erik Sahai, Igor Astsaturov, Edna Cukierman, David G. DeNardo, Mikala Egeblad, Ronald M. Evans, Douglas Fearon, Florian R. Greten, Sunil R. Hingorani, Tony Hunter, Richard O. Hynes, Rakesh K. Jain, Tobias Janowitz, Claus Jorgensen, Alec C. Kimmelman, Mikhail G. Kolonin, Robert G. Maki, R. Scott Powers, Ellen Puré, Daniel C. Ramirez, Ruth Scherz-Shouval, Mara H. Sherman, Sheila Stewart, Thea D. Tlsty, David A. Tuveson, Fiona M. Watt, Valerie Weaver, Ashani T. Weeraratna, Zena Werb

**Affiliations:** 1 The Francis Crick Institute, London, UK; 2 Marvin and Concetta Greenberg Pancreatic Cancer Institute, Fox Chase Cancer Center, Philadelphia, PA USA; 3 Cancer Biology Program, Marvin & Concetta Greenberg Pancreatic Cancer Institute, Fox Chase Cancer Center, Philadelphia, PA USA; 4 Division of Oncology, Washington University Medical School, St Louis, MO USA; 5 Cold Spring Harbor Laboratory, Cold Spring Harbor, NY USA; 6 Gene Expression Laboratory, Salk Institute for Biological Studies, La Jolla, CA USA; 7 Howard Hughes Medical Institute, Salk Institute for Biological Studies, La Jolla, CA USA; 8 Weill Cornell Medicine, New York, NY USA; 9 Institute for Tumor Biology and Experimental Therapy, Georg-Speyer-Haus, Frankfurt, Germany; 10 Frankfurt Cancer Institute, Goethe University Frankfurt, Frankfurt, Germany; 11 Fred Hutchinson Cancer Research Center, Seattle, WA USA; 12 Molecular and Cell Biology Laboratory, Salk Institute for Biological Studies, La Jolla, CA USA; 13 Koch Institute for Integrative Cancer Research, Massachusetts Institute of Technology, Cambridge, MA USA; 14 Edwin L Steele Laboratories, Department of Radiation Oncology, Massachusetts General Hospital, Harvard Medical School, Boston, MA USA; 15 Northwell Health Cancer Institute, New Hyde Park, NY USA; 16 Cancer Research UK Manchester Institute, University of Manchester, Nether Alderley, UK; 17 Department of Radiation Oncology, Perlmutter Cancer Center, New York University Medical Center, New York, NY USA; 18 Brown Foundation Institute of Molecular Medicine, The University of Texas Health Sciences Center at Houston, Houston, TX USA; 19 Northwell Health Cancer Institute, New York, NY USA; 20 Department of Pathology, Stony Brook University, Stony Brook, NY USA; 21 Department of Biomedical Sciences, School of Veterinary Medicine, University of Pennsylvania, Philadelphia, PA USA; 22 Zucker School of Medicine at Hofstra/Northwell Health System, New York, NY USA; 23 Department of Biomolecular Sciences, The Weizmann Institute of Science, Rehovot, Israel; 24 Department of Cell, Developmental & Cancer Biology, Oregon Health & Science University, Portland, OR USA; 25 Department of Cell Biology and Physiology, Department of Medicine, ICCE Institute, Siteman Cancer Center, Washington University School of Medicine, St Louis, MO USA; 26 UCSF Helen Diller Comprehensive Cancer Center, San Francisco, CA USA; 27 Department of Pathology, UCSF, San Francisco, CA USA; 28 Centre for Stem Cells and Regenerative Medicine, King’s College London, Guy’s Hospital, London, UK; 29 Center for Bioengineering and Tissue Regeneration, Department of Surgery, University of California, San Francisco, San Francisco, CA USA; 30 Sidney Kimmel Cancer Center, Johns Hopkins School of Medicine, Baltimore, MD USA; 31 Department of Anatomy, University of California, San Francisco, San Francisco, CA USA; 32 Present Address: Abramson Cancer Center, University of Pennsylvania, Philadelphia, PA USA

**Keywords:** Cancer microenvironment, Metastasis, Extracellular matrix, Cancer therapy, Cancer therapeutic resistance

## Abstract

Cancer-associated fibroblasts (CAFs) are a key component of the tumour microenvironment with diverse functions, including matrix deposition and remodelling, extensive reciprocal signalling interactions with cancer cells and crosstalk with infiltrating leukocytes. As such, they are a potential target for optimizing therapeutic strategies against cancer. However, many challenges are present in ongoing attempts to modulate CAFs for therapeutic benefit. These include limitations in our understanding of the origin of CAFs and heterogeneity in CAF function, with it being desirable to retain some antitumorigenic functions. On the basis of a meeting of experts in the field of CAF biology, we summarize in this Consensus Statement our current knowledge and present a framework for advancing our understanding of this critical cell type within the tumour microenvironment.

## Abstract (web summary)

This Consensus Statement highlights the importance of cancer-associated fibroblasts in cancer biology and progression, and issues a call to action for all cancer researchers to standardize assays and report metadata in studies of cancer-associated fibroblasts to advance our understanding of this important cell type in the tumour microenvironment.

## Introduction

Cancer arises from mutations accruing within cancer cells, but both disease progression and responses to therapy are strongly modulated by non-mutant cells within the tumour microenvironment. The past few years have witnessed a great expansion in research into cancer-associated fibroblasts (CAFs). These cells modulate cancer metastasis through synthesis and remodelling of the extracellular matrix (ECM) and production of growth factors, and influence angiogenesis, tumour mechanics, drug access and therapy responses. More recently, there has been a growing appreciation of the ability of CAFs to modulate the immune system. Targeting CAFs, by altering their numbers, subtype or functionality, is being explored as an avenue to improve cancer therapies. However, research in this area faces numerous challenges — not least because CAFs can have both protumorigenic and antitumorigenic effects. This Consensus Statement follows a recent Banbury Center meeting at Cold Spring Harbor Laboratory (New York, USA) held in March 2019, which focused on CAF biology and therapeutic opportunities and included an open discussion to identify the challenges facing CAF research and suggest ways forward (Box 1). On the basis of this, we, as an international group of cancer researchers and clinician scientists, herein present the current state of CAF research, summarize the challenges ahead and present both methodological advice and conceptual suggestions to provide the necessary framework to advance the field.

Box 1 Generation of this Consensus Statement This Banbury Center meeting convened experts to discuss our current understanding of cancer-associated fibroblast (CAF) biology, with an emphasis on optimizing new approaches being developed to probe the fundamental properties of CAFs, and medical applications of CAF targeting. Following the introductory remarks, the idea of summarizing the outputs from the meeting in a Consensus Statement was proposed and unanimously approved. An open discussion with all meeting participants was held on the final day of the meeting to collate ideas about what the Consensus Statement should contain and how it should be structured. A draft statement was then circulated to all authors for feedback and refinement, leading to agreement with the views expressed in this Consensus Statement.

## What is a fibroblast?

The definition of a fibroblast is surprisingly tricky^1,2^. The embryonic origin of most fibroblasts is from the primitive mesenchyme that develops out of the mesoderm following gastrulation^3^, with a smaller subset of fibroblasts also derived from the neural crest, which is part of the ectoderm^4^. This embryonic origin is shared by other mesenchymal lineages, including adipocytes, chondrocytes and osteoblasts. The difficulty in defining fibroblasts results largely from the lack of unique markers that are not expressed in any other cell types. The result is that in practical terms, fibroblasts are often defined by a combination of their morphology, tissue position and lack of lineage markers for epithelial cells, endothelial cells and leukocytes. Vimentin and platelet-derived growth factor receptor-α (PDGFRα) are sometimes used but typically alongside other criteria such as cell shape and location. Markers for fibroblast subtypes also exist, including α-smooth muscle actin (αSMA; also known as ACTA2) and fibroblast activation protein (FAP)^5,6^, with the subset of fibroblasts expressing the latter playing roles in bone and fat homeostasis. Recent work is beginning to trace the lineage of fibroblasts from the earliest stage of mesenchyme specification through to the adult. This has already highlighted distinct subsets of dermal fibroblasts and is starting to provide more precise combinations of markers with which to define fibroblasts^7,8^. However, the links between lineage commitment in early development and the fibroblast subsets found in the adult remain, for the most part, to be determined.

In normal development and physiology, fibroblasts are the major producers of connective tissue ECM, with emerging data indicating that this function is modified with age^9,10^. They also play a key role in tissue repair and become activated following tissue damage^11^. During wound healing they can produce transforming growth factor-β (TGFβ) and acquire a highly contractile phenotype associated with the expression of αSMA^12^. In this state, fibroblasts are termed ‘myofibroblasts’. Both in normal homeostasis and following injury they participate in crosstalk with adjacent epithelia, with numerous studies documenting an ability to influence local epithelial stem cell behaviour^13,14^. They can also promote angiogenesis via the production of vascular endothelial growth factor A (VEGFA)^15^ and coordinate the function of the immune system via the production of chemokines and cytokines, although it should be noted that there is heterogeneity in the cytokines produced by different fibroblasts^16–20^. Fibroblasts also play a structural role within the immune system; fibroblastic reticular cells (FRCs) within lymph nodes generate ECM conduits for the transit of potential antigens and serve as migration ‘highways’ for leukocytes^21^. This allows effective immune surveillance. In addition, they promote immune tolerance by the expression and presentation of normally tissue-restricted antigens^22^. Emerging work is revealing complex crosstalk between fibroblastic cells and epithelial cells in exocrine organs. For example, stellate cells are a distinctive type of fibroblast found in the liver and pancreas that store lipid droplets and particular derivatives of retinoic acid. The balance between quiescence and activation of stellate cells is regulated by the vitamin D receptor, deletion of which leads to spontaneous liver and pancreas fibrosis^23^, with further work indicating that stellate cells play a broader role in metabolic homeostasis^24–26^. Thus, fibroblasts are not simply producers of ECM but play key roles in communicating with many other cell types during both normal tissue homeostasis and repair.

## What is a CAF?

To first consider how CAFs are generated, it is important to try to define CAFs. Many of the same issues arising for normal fibroblasts also apply to CAFs. When analysing tissue biopsy samples, the simplest view is that cells negative for epithelial, endothelial and leukocyte markers with an elongated morphology and lacking the mutations found within cancer cells might be considered CAFs. The latter point is important because it excludes cancer cells that have undergone a profound epithelial to mesenchymal transition (EMT), although such cells are likely to be of considerable importance and warrant studying in their own right. In practice, lineage exclusion is typically combined with positivity for a mesenchymal marker, often vimentin; however, this may not be sufficient to exclude other mesenchymal lineages such as pericytes or adipocytes. Early experimental studies indicated that such cells cultured from tumours have distinctive properties compared with normal fibroblasts^27^. In practice, any mesenchymal cell cultured from a tumour that complies with the criteria described above is considered a CAF. Nevertheless, as discussed in the section entitled ‘Challenges and recommendations’, how durable CAF subsets and phenotypes remain once fibroblasts are isolated and cultured warrants further investigation.

## What is the origin of CAFs?

The lack of precision around fibroblast-specific markers poses a challenge when one is considering the origin of CAFs. When the markers of both normal tissue-resident fibroblasts and CAFs are ill-defined, it becomes very hard to propose hypotheses regarding the precise cell of origin of CAFs. To partially circumvent this problem, many studies have documented the changes in the fibroblastic component of carcinomas as they progress from hyperplasia, through adenoma or in situ lesion, to frank carcinoma in patients^28^. These studies using human tissue observe progressive changes in the fibroblastic stroma. In many cases, the initial apparent expansion of fibroblasts precedes the conversion to malignancy, and fibroblasts are often observed circumscribing early or premalignant lesions^29,30^. The gradual nature of the transitions observed has given rise to the view that the majority of stromal fibroblasts initially originate from local fibroblasts that experience some form of tissue dysfunction^31,32^. The expansion of stromal fibroblast number could result from proliferation, which has been experimentally observed in tumours, albeit with low frequency^33^. This process has been termed ‘stromagenesis’, with the implication that it proceeds alongside and is coupled to tumorigenesis^34^. Furthermore, experimental studies and observations of early lesions encircled by fibroblasts support the idea that the initial fibroblast response can be tumour suppressive^35,36^, with subsequent events in stromagenesis generating protumorigenic fibroblasts. It is currently difficult to explore this hypothesis in human tissue biopsy samples because longitudinal sampling of the same lesion through disease progression is rarely possible and, even when it is, the conversion between cell states cannot be directly tracked.

To shed more light on the origin of CAFs, many researchers have turned to mouse models in which cells can be irreversibly labelled using transgenic techniques and well-characterized models of disease progression are available. These typically use tissue-restricted expression of the Cre recombinase in mice that also contain a reporter gene that becomes irreversibly active in cells that express Cre. Importantly, the active reporter will be inherited by all daughter cells and will continue to be expressed even if the Cre recombinase is not^37^. However, the lack of fibroblast-restricted markers causes problems when one is selecting a promoter to drive the expression of the Cre recombinase. This is exemplified by the divergent phenotypes observed in a colitis model of stromal knockout of *Ikkb* (inhibitor of nuclear factor-κB (NF-κB) kinase subunit-β) depending on whether a collagen type I α2 chain (*Col1a2*) or collagen type V α1 chain (*Col5a1*) Cre driver was used^38,39^. The most widely used fibroblast drivers and their caveats are detailed in the section entitled ‘Challenges and recommendations’. This approach can also be used to explore hypotheses including the conversion of adipocytes, pericytes, endothelial cells and bone marrow-derived mesenchymal stem cells (MSCs) into CAFs. The injection of bone marrow-derived MSCs into tumour-bearing mice has demonstrated that they can become CAFs^40^, with more recent studies supporting the MSC origin of PDGFRα^–^ CAFs^41^. Adipocyte conversion into CAFs has been reported by several groups, although it does not appear to be a universally applicable phenomenon across different tumour types^42–45^. The reduction or absence of adipocytes in pathological tissue could also result from activated fibroblasts interfering with adipocyte differentiation^46^. In situations where adipocytes remain, they can engage in crosstalk with cancer cells and provide metabolic support independently of conversion into CAFs^47^. Evidence for pericyte conversion into CAFs is relatively sparse^48^, with tumorigenesis studies that targeted pericytes specifically not revealing large-scale differences in the tumour microenvironment.

Ultimately, lineage tracing studies in mice remain hampered by the lack of highly specific Cre drivers for normal fibroblasts, difficulties of combining lineage tracing with genetically engineered models of mouse tumours that are also driven by Cre recombinase, the suboptimal nature of cell line-based tumour models and the lack of incentives to report negative data in such studies. Currently, it is also unclear whether individual CAF populations are preserved across tissues and species. While single-cell sequencing indicates common traits are preserved^49–51^, it will become increasingly pressing to define common and specific effects of CAFs. Techniques that provide spatial resolution, such as highly multiplexed antibody-based staining and multiplexed nucleic acid in situ hybridization, will also have a role to play in determining whether CAF subtype is strongly influenced by spatial location within the tumour. Together, these factors mean that definitive conclusions on the origin of CAFs are hard to reach. The consensus is that most CAFs likely result from the activation of local tissue-resident fibroblasts but that there are clear examples of alternative origins.

## How are CAFs generated?

**Fig. 1:**
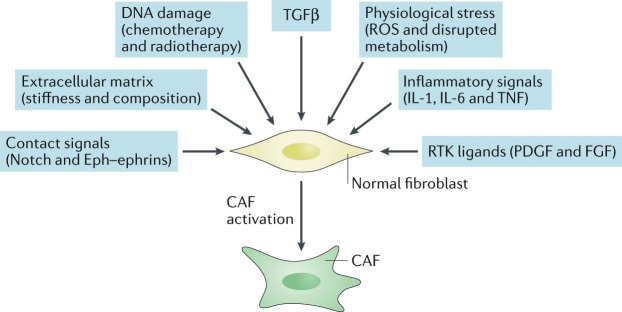
Diverse mechanisms of cancer-associated fibroblast activation. This schematic highlights the multiple mechanisms that can contribute to cancer-associated fibroblast (CAF) activation. FGF, fibroblast growth factor; PDGF, platelet-derived growth factor; ROS, reactive oxygen species; RTK, receptor tyrosine kinase; TGFβ, transforming growth factor-β; TNF, tumour necrosis factor.

The studies described in the previous section aimed to document which cells give rise to CAFs but do not provide a mechanism for their conversion. Given the prominent role fibroblasts play in coordinating the wound repair response in skin, it is plausible that key CAF traits correspond to the normal physiological role fibroblasts play. Well-established activating signals for fibroblasts include TGFβ family ligands and the lipid mediator lysophosphatidic acid^52–54^, which promote the activity of the SMAD transcription factors and serum response factor (SRF), respectively, and converge to drive expression of the activated fibroblast marker αSMA as well as increase the activity of the contractile cytoskeleton^6^ (Fig. 1). Contact between cancer cells and fibroblasts can promote the CAF phenotype in breast cancer through Notch signalling^55^; however, this mechanism is unlikely to be universal as loss of Notch signalling can promote CAF phenotypes in squamous cell carcinoma^56^. Various inflammatory modulators can promote CAF activation, with interleukin-1 (IL-1) acting through NF-κB and IL-6 acting primarily on signal transducer and activator of transcription (STAT) transcription factors^57,58^. Crosstalk and positive feedback involving Janus kinase (JAK)–STAT signalling, the contractile cytoskeleton and alterations in histone acetylation further promote CAF activation^59,60^. Physical changes in the ECM are also capable of activating CAFs^53,61–64^. In vitro studies have shown that fibroblast stretching, which may result from the hyperproliferation of transformed epithelial cells, can activate SRF-driven transcription and Yes-associated protein 1 (YAP1)–TEAD-driven transcription^53,54,65,66^. These transcription factors cooperate to drive the expression of a wide-range of genes associated with CAFs, including the genes encoding connective tissue growth factor (*CTGF*; also known as *CCN2*) and cysteine-rich angiogenic inducer 61 (*CYR61*; also known as *CCN1*)^54^. Furthermore, matricellular molecules, such as CTGF and CYR61, and the contractile cytoskeleton cooperate to increase tissue stiffness, which further drives SRF-dependent and YAP1-dependent transcriptional programmes, locking CAFs into a self-sustaining positive-feedback loop^53^. Physiological stress is also another factor contributing to stromagenesis. Heat shock factor 1 (HSF1), which responds in part to protein misfolding, is required for the generation of CAFs^67,68^. Physiological and genomic stresses can also trigger changes in fibroblasts. Double-stranded DNA breaks can promote the production of IL-6 and the TGFβ family ligand activin A^69,70^. In some cases, these triggers cause fibroblasts to enter a non-proliferative state termed ‘senescence’^71^, which is distinct from the phenotype of an aged fibroblast. There is clear overlap between the secretome of senescent fibroblasts and CAFs, with high levels of IL-6 production being common to both, and senescent fibroblasts have been found in the microenvironment of some tumours^72^. The non-proliferative nature of senescent cells makes it unlikely that they are a major contributor to the abundant stromal fibroblasts observed in desmoplastic tumours^73^. Nonetheless, it remains possible that CAFs and senescent fibroblasts share some transcriptional regulatory mechanisms^74,75^. Furthermore, even if senescent fibroblasts are a minor component of the tumour microenvironment, experimental analysis suggests that their elimination can have substantial consequences for disease relapse^71^.

In addition to considering tumour cells as the direct source of cues that generate CAFs, signals from other cells within the tumour microenvironment may also instruct CAF function; for example, granulin produced by macrophages promotes the activation of a fibrotic environment in liver metastases^76,77^. Such mechanisms that do not directly depend on the presence of cancer may contribute to the protumorigenic environments found in inflammatory conditions that are linked to increased cancer risk. In addition, cancer therapies, including conventional chemotherapies, radiotherapy and targeted agents, can promote the generation of CAFs and modulate their functionality. These changes can aid the development of therapy resistance^78–80^ and contribute to undesirable side effects^80^. Being able to mitigate these events is another potential appeal of CAF-directed therapies.

## The expanding range of CAF functions

**Fig. 2:**
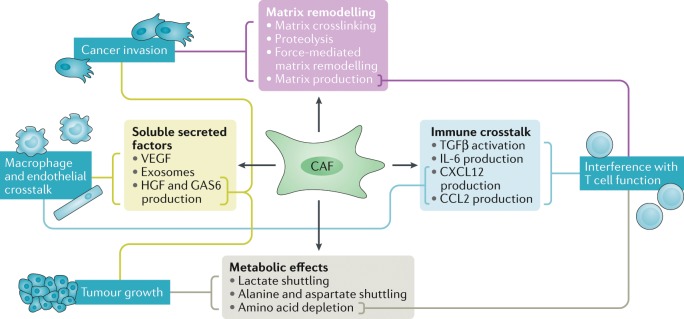
Summary of cancer-associated fibroblast functions and the mechanisms by which they are achieved. Dark blue text boxes indicate the biological functions being regulated, with light blue, green, purple and grey text boxes indicating the processes and mechanisms leading to the control of function. Lines connect mechanisms to functions. Both matrix remodelling and the production of soluble factors contribute to increased tumour cell invasion. Soluble factors also contribute to changes in tumour growth and the immune microenvironment, which is also affected by the altered metabolic state of the tumour. CAF, cancer-associated fibroblast; CCL2, CC-chemokine ligand 2; CXCL12, CXC-chemokine ligand 12; IL-6, interleukin-6; GAS6, growth arrest-specific protein 6; HGF, hepatocyte growth factor; TGFβ, transforming growth factor-β; VEGF, vascular endothelial growth factor.

The functions of CAFs have been determined using a variety of strategies, ranging from reductionist cell culture experiments and mouse models to correlative associations in large patient cohorts. These approaches have revealed a diverse array of functions (Fig. 2). The relative ease of culturing CAFs and matched normal fibroblasts from patient material has greatly facilitated mechanistic delineation of CAF functions. CAFs are perhaps the most effective cell within the tumour microenvironment at depositing and remodelling the ECM. This depends on RHO and RAB GTPase-mediated control of integrin-mediated adhesions and the actomyosin cytoskeleton^81–83^ and is linked to downregulation of the transmembrane receptor CD36 (also known as platelet glycoprotein 4)^84^. CAFs also produce matrix-crosslinking enzymes and, together with force-mediated ECM remodelling (reviewed in detail^85,86^), these contribute to the increased stiffness of tumour tissue^87–89^. Although chemical crosslinks are not readily reversed, the production of matrix proteases allows the tumour matrix to be remodelled, and this can lead to the generation of permissive tracks that allow cancer cell invasion^81^. Contact-mediated Eph–ephrin signalling further influences cancer cell migration^90^. In addition to promoting local invasion, CAFs are able to boost metastasis in experimental models, and this correlated with their ability to remodel the ECM^91–93^. Once cancer cells have disseminated, the de novo activation of fibroblasts at secondary sites favours the establishment of macrometastases via multiple mechanisms, including the production of matrix components such as tenascin and periostin that provide supporting signals to the cancer cells^94,95^. These molecules boost WNT signalling^94^, which may link to the role of some fibroblasts in normal physiology in regulating stem cell niches that are rich in WNT ligands^96,97^. More recently, changes in ECM organization have been shown to influence the migration of infiltrating leukocytes, which has implications for the immune surveillance of tumours^98^.

The alterations in matrix production and tumour mechanics that result in a large part from the action of CAFs have complex consequences for tumours. Increased tissue stiffness triggers prosurvival and proproliferation signalling in cancer cells^99^. Increased mechanical stress can collapse blood vessels, leading to hypoxia, thereby promoting more aggressive cancer phenotypes, and reducing drug delivery^89,100–105^. Altered tissue mechanics are also likely to play a role in cancer development and premalignant disease; this is evidenced by the links between mammographic density and breast cancer incidence^84^. Targeting the interplay between CAFs and the mechanical properties of tumours for patient benefit is currently being explored (see Table 1).

**Table 1:** Current cancer-associated fibroblast clinical trial activity

Target	Name	Drug or biologic	Mechanism	Current status
***Interference with CAF activation***				
FGFR	JNJ-42756493	Small-molecule inhibitor	Prevents CAF activation	Phase I and phase II trials under way^170^
Hedgehog	IPI-926 (saridegib) and vismodegib	Small-molecule inhibitor	Reduces CAF activation	Clinical trials ongoing; some reported lack of efficacy^169,171^
***Interference with CAF activation and CAF action***				
TGFβ	Various, including galunisertib	Both blocking Abs and small-molecule receptor inhibitors	Prevents CAF activation and immunosuppression	Phase I, phase II and phase III trials under way^172,173^
Angiotensin receptor	Losartan	Small-molecule inhibitor	Reduces collagen and hyaluronan levels	Phase II trial completed; randomized trial ongoing^174,175^
***Interference with CAF action***				
CXCR4	AMD3100	Small-molecule inhibitor	Prevents signalling from CAFs to immune cells	Clinical trials ongoing^176^
ROCK	AT13148	Small-molecule inhibitor	Reduces contractility	Phase I trial completed^177^
FAK	Defactinib (VS-6063, PF-04554878)	Small-molecule inhibitor	Reduces signalling downstream of integrins	Clinical trials ongoing^178^
LOXL2	Simtuzumab (GS 6624)	Blocking Ab	Anticrosslinking	Preclinical and fibrosis trials^179^
CTGF	FG-3019	Blocking Ab	Blocks binding to receptors, including integrins	Early-phase clinical trials ongoing
Hyaluronic acid	PEGPH20 (PVHA)	Pegylated enzyme	ECM degradation to increase the access and efficacy of cytotoxic therapies and immunotherapies	Phase III trial complete, awaiting final analysis^180,181^
FAP-expressing cells	Various, including PT630 and RO6874281	Blocking Abs (sibrotuzumab I (ref.^182^), molecular radiotherapy, inhibitors (PT630) or an Ab–IL-2 fusion (RO6874281)	Blocks FAP^+^ CAF function, promoting T cell function	Phase I and phase II trials under way^183^
***CAF normalization***				
Vitamin A metabolism	ATRA	Vitamin A metabolite	‘Normalizes’ stellate cells	Clinical trials ongoing^184,185^
Vitamin D receptor	Paricalcitol	Small-molecule agonist	‘Normalizes’ stellate cells	Clinical trial started^186^

Ab, antibody; ATRA, all-*trans* retinoic acid; CAF, cancer-associated fibroblast; CTGF, connective tissue growth factor; CXCR4, CXC-chemokine receptor 4; ECM, extracellular matrix; FAK, focal adhesion kinase; FAP, fibroblast activation protein; FGFR, fibroblast growth factor receptor; IL-2, interleukin-2; LOXL2, lysyl oxidase-like 2; ROCK, RHO kinase; TGFβ, transforming growth factor-β.

CAFs are also a substantial source of growth factors, cytokines and exosomes that can promote tumour growth and modulate therapy responses^27,106–108^. The production of TGFβ, leukaemia inhibitory factor (LIF), growth arrest-specific protein 6 (GAS6), fibroblast growth factor 5 (FGF5), growth differentiation factor 15 (GDF15) and hepatocyte growth factor (HGF) promotes invasive and proliferative behaviour in cancer cells^52,109–112^. In addition, HGF has been implicated in mediating resistance to BRAF-targeted therapies by providing an alternative BRAF-independent mechanism for ERK–MAPK activation^113^.

The secretome of CAFs also influences other components of the tumour microenvironment. VEGF expression by stromal cells can drive angiogenesis^15,114^. Numerous cytokines and chemokines are produced by CAFs, and these act on a range of leukocytes, including CD8^+^ T cells, regulatory T (T_reg_) cells and macrophages, with both immunosuppressive and immunopromoting consequences^115^. However, the consensus is that the predominant effect of CAFs is immunosuppressive with IL-6, CXC-chemokine ligand 9 (CXCL9) and TGFβ having well-established roles in reducing T cell responses^116^. More recently, antigen cross presentation by CAFs has been observed^117^, and this may lead to CD4^+^ T cell activation and suppression of CD8^+^ T cells^118^. Clinical analysis further supports an inverse association between CAFs and CD8^+^ T cells^119^. IL-6 may also promote immunosuppression via systemic effects on metabolism^120^. Interference with the action of CXCL12 produced by CAFs promotes T cell-mediated tumour control^16,121,122^, and targeting focal adhesion kinase (FAK) in cancer cells concomitantly reduces stromal fibroblast activation and the development of an immunosuppressive environment^123^. However, the situation with tumour necrosis factor (TNF) produced by CAFs is more nuanced; the tumour-promoting immunosuppressive activity of FAP^+^ fibroblasts is associated with suppression of TNF signalling, yet TNF is also able to drive fibroblast activation in certain contexts^16,124,125^.

The exchange of metabolites and amino acids between cancer cells and CAFs is an additional avenue by which stromal fibroblasts interact with tumour cells^126–129^. Autophagy in stromal fibroblasts can generate alanine, which is subsequently used by pancreatic ductal adenocarcinoma (PDAC) cells to fuel the tricarboxylic acid (TCA) cycle^126,130,131^. Furthermore, metabolic dysregulation of CAFs may also be coupled to altered immunoregulation, possibly through IL-6 production or depletion of immunomodulating amino acids^128,132^.

## CAF heterogeneity and plasticity

The large array of functions attributed to CAFs in a range of model systems poses the question of whether a single type of CAF simultaneously performs all these functions or whether there is subspecialization of CAFs and possibly switching between distinct functional states. Overwhelming evidence now points to a degree of specialization among CAFs, which may reflect the increasingly appreciated specialization of normal fibroblasts^19,50^. This is informed by the increasing array of functional assays combined with the emergence of single-cell technologies, including single-cell RNA sequencing^48,49,133^. New analyses are being reported at an impressive rate, and the field is in a state of flux. Nonetheless, there is a recurrent observation of distinct CAFs exhibiting either a matrix-producing contractile phenotype or an immunomodulating secretome — often termed ‘myoCAFs’ and ‘iCAFs’, with the prefixes alluding to a myofibroblast phenotype and regulation of inflammation, respectively. In pancreatic cancer, CAFs most proximal to the cancer cells exhibit a myoCAF phenotype, with high TGFβ-driven αSMA expression and a contractile phenotype^33^. More distal CAFs express higher levels of IL-6 and are labelled iCAFs. The apparent exclusivity of the two phenotypes can be explained by TGFβ-mediated suppression of the IL-1 receptor, which is responsible for driving NF-κB signalling and subsequent IL-6 expression^20^. Breast cancer also shows divergent CAF phenotypes, with the primary discriminating marker being FAP. FAP-high fibroblasts are correlated with T_reg_ cell-mediated immunosuppression and a poor outcome^119^, which is broadly consistent with the tumour rejection observed following the ablation of FAP^+^ fibroblasts in experimental systems^16^. However, FAP^+^ fibroblasts should not be viewed as solely immune modulating, as their targeting with chimeric antigen receptor (CAR) T cells leads to reduced matrix deposition^134^. Another study reported an NF-κB-driven subset of CAFs expressing GPR77 and CD10, which promote ‘stemness’ and chemoresistance within breast cancer cells^135^. In the long term, it will be important for researchers to coalesce around a consensus for CAF subtypes and nomenclature (discussed in more detail later). Improvements in multiplexed immunohistochemistry that allow the analysis of multiple markers simultaneously and more quantitative methods for determining relative degrees of marker expression should aid reproducible evaluation of CAF subtypes.

The issue of CAF heterogeneity raises additional questions; including whether CAF subtypes might interconvert or whether they are more stable, possibly because they are instructed by oncogenic or tumour suppressor mutations within cancer cells. Knowledge in this area is currently emerging. Work in PDAC has shown how *KRAS* mutation or different p53 mutational status can influence CAFs^111,136^. Mutant p53 drives TNF production by cancer cells, leading to enhanced matrix remodelling and perlecan expression by CAFs^136^. However, such studies do not preclude additional non-genetic factors influencing CAF subtype. Indeed, CAFs isolated from mouse PDAC can be switched from the αSMA-high and IL-6-producing states through manipulation of TGFβ and IL-1 signalling, arguing for considerable plasticity in fibroblast states^20^. Furthermore, the responsiveness of matrix production by fibroblasts and the αSMA promoter to a range of extracellular cues, including substrate stiffness, supports the idea that the αSMA-high, matrix producing-high state is reversible^63,64,137–139^. Once again, irreversible lineage marking approaches in mouse models should be informative in addressing the interconvertibility of different CAF subtypes, and improved understanding of the epigenetic regulation of CAF states should shed further light on the stability of CAFs.

## Targeting CAFs for clinical benefit

Many patient studies have documented how either CAF number or CAF function is linked to outcome^140–142^, and thus being able to target CAFs would represent an appealing addition to the suite of anticancer therapies. Further targeting mechanisms, such as TGFβ signalling, that activate CAFs or emanate from CAFs to modulate the tumour phenotype are being intensively explored^143,144^. There is already much activity in the area of CAF targeting — summarized in Box 2, Table 1 and detailed reviews^145,146^. However, the breadth of CAF functions and possible interconvertibility of subtypes poses a challenge for the field, with preclinical studies suggesting that the non-specific targeting or deletion of stromal fibroblasts may not enhance tumour control^35,36^. Thus, patient benefit might require targeting of CAF subtypes or reprogramming of CAFs to either a normal fibroblast or an antitumorigenic CAF phenotype. This highlights the importance of defining CAF subtypes and their inter-relationships. One appealing strategy is to make CAFs more ‘normal’. An example of this approach is provided by the targeting of the vitamin D receptor in pancreatic cancer. Treatment with a vitamin D receptor ligand caused activated stellate cells to revert to a more quiescent state and reduced disease aggressiveness^23,147^ (see Table 1). Therefore, it is important to delineate whether individual fibroblast populations represent ‘states’ and are therefore interconvertible or whether distinct ‘lineage-restricted’ effects exist as this may dictate a different therapeutic approach. The functional contribution of CAFs to tumour biology is also typically assumed to be preserved across tumour types, but this remains to be demonstrated, and care will be needed when one is extrapolating between different tumour types.

In practice, achieving clinical benefit may not necessarily require elimination or reprogramming of CAFs, but could be achieved by blocking signals coming from the CAFs. For example, targeting CXCL12 signalling could be considered to be targeting CAFs as they are the major source of the chemokine in many tumours^121^. Similarly, targeting ECM components and downstream signalling represents a means of interfering with CAF–cancer cell communication. Indeed, many existing therapies influence CAF–cancer cell communication and already modulate how CAFs affect cancer cells. As mentioned earlier, BRAF inhibitors can activate stromal fibroblasts and thereby promote a compensatory mechanism for activating ERK–MAPK in cancer cells^78^. Many of the expanding range of receptor tyrosine kinase inhibitors have some activity on FGF and PDGF receptors that can drive fibroblast function^148,149^. This is exemplified by the repurposing of nintedanib, which was originally developed with oncology in mind, for treatment of idiopathic pulmonary fibrosis^150^. Finally, both conventional DNA damaging chemotherapy and radiotherapy can trigger changes in CAF biology, with fibrosis being a common late side effect of radiotherapy^151^. These data argue that more studies to assess the extent to which responses to therapies might be influenced by altered CAF biology are warranted.

Box 2 Cancer-associated fibroblast clinical trial activity Cancer-associated fibroblasts (CAFs) are increasingly viewed as a target that could be manipulated for therapeutic benefit in patients with cancer. There are now many clinical trials involving CAF-targeting agents in combination with existing therapies. The underlying rationale is that by targeting CAFs there will be improvements in the access of either conventional therapies or T cells to the tumour. In some cases, new strategies are being developed to target fibroblasts specifically (for example, fibroblast activation protein (FAP) ligands coupled to cytotoxic drugs^165^). In other cases, crosstalk between cancer cells and fibroblasts is targeted (for example, Hedgehog pathway inhibition^166^), or existing compounds are found to have a strong influence on CAF functions and are repurposed as anti-stromal drugs (for example, losartan is primarily used to treat high blood pressure but also modulates the tumour extracellular matrix^100,101,167,168^). Table 1 outlines ongoing clinical trial activity in these areas.

**Possible lessons from targeting the Hedgehog pathway in pancreatic cancer**

The clinical trial designed to recapitulate the advantageous effect of Hedgehog pathway inhibition in mouse models of pancreatic ductal adenocarcinoma (PDAC)^166^ failed to show such benefit and paradoxically reported decreased patient survival in combination with gemcitabine chemotherapy (NCT01130142)^169^. The details of this trial have not yet been described, and subsequent preclinical studies that suppressed in the long term the CAF subset known to be Hedgehog responsive also demonstrated more rapid PDAC progression^35,36^. One possible explanation for this discordance between the first mouse experiments and the latter ones, and the failed clinical trial, is that CAFs are interconvertible from Hedgehog responsive to Hedgehog non-responsive over time, and this should be considered in the design of new studies. Indeed, it is now common practice for clinical trials to evaluate the numbers of different classes of T cells, and it would be an important advance if data on CAF numbers and subtypes, at least based on some key markers, were also captured.

## Challenges and recommendations

### An emerging framework for nomenclature

A key challenge now facing researchers of CAF biology is nomenclature (Box 3). Ideally a system should be simple enough to allow it to be used by the wider cancer and stromal biology communities but not so dogmatic and constrictive that it masks subtle variations in function and markers. In addition, it must have flexibility to incorporate fibroblast subtypes that are only currently being revealed by single-cell transcriptomics and mass cytometry methods. Although our view was that it is too soon for definitive nomenclature to be established, the consensus was that the main determinant of CAF categorization should be function, informed primarily by direct experimental evidence and, in some cases, robust clinical correlation analyses. These categories should then be linked to markers, ideally cell surface markers, so that they can be further interrogated in analyses that might not be compatible with functional testing. A sensible starting point for such a classification would be the reiteration that activated fibroblasts can adopt a high matrix producing and remodelling state — analogous to the myofibroblast in other pathologies. This is linked to high levels of TGFβ signalling and αSMA expression^6^. It will also be necessary to include immunomodulation into CAF categories. Although most studies have suggested an immunosuppressive role of CAFs^121^, it should be left open that CAFs could promote immune-mediated tumour surveillance. Indeed, the function of FRCs in lymph nodes is to make possible an effective T cell-mediated immune response^21^. Scope should also be left for antigen presentation, the metabolic state of fibroblasts, and their lineage history to be incorporated into any nomenclature.

Box 3 Key recommendations

- Adoption of a simple, non-constrictive nomenclature based on cancer-associated fibroblast (CAF) function
- Relate fibroblast markers to function while avoiding dogmatic schemes that do not account for diversity of fibroblast states
- Determine the lineage relationship of different CAF subtypes
- Prioritize identification of strategies that can reprogramme CAFs rather than ablate them
- Increased reporting of CAF metadata in experimental studies, including clinical features of the tumour, CAF marker expression in the original tumour, short tandem repeat profile, culture conditions and passage number, and immortalization
- Recording of CAF numbers in clinical studies and trials, starting with reporting of α-smooth muscle actin (αSMA) and fibroblast activation protein (FAP) staining

### Robust and standardized methods for detecting CAFs in tissue

Progress in translational studies will require accurate recording of CAF numbers and subtype within clinical samples. Clinical studies that target either CAFs or CAF-associated functions must include measurement of CAFs in their design. More generally, our consensus was that CAF metrics should be recorded even in studies that do not have CAFs as their focus, for example in immuno-oncology trials. This will depend on high-quality antibodies against CAF marker proteins, which in many cases are lacking. While reliable αSMA antibodies are available, antibodies against putative CAF subtype markers often require painstaking optimization, and this hampers their adoption in clinical pathology laboratories. The technology around multiplexed mRNA probes is developing rapidly and, in the long term, this might provide a better and more flexible solution than antibody-based methods. Furthermore, researchers are aware of caveats in studies that involve dissociation of tumour tissue, for example those using cytometry by time of flight (CyTOF) and single-cell RNA sequencing. Detaching fibroblasts from tissue typically requires more aggressive methods than for leukocytes, and there is risk that fibroblasts are substantially under-represented in studies optimized for leukocyte biology^152^.

### Measuring CAF functions in vitro and in vivo

The diversity of CAF function is reflected in the wide range of assays used to assess CAF function. While the breadth of assays is necessary, it presents a challenge when one is interpreting the literature. In this subsection, we review the main assays used and highlight key points regarding interpretation of their results.

The function of CAFs can be directly investigated in vitro. Given the ability of both serum and stiff substrates to activate fibroblasts, attention should be paid to the culture conditions used, with lower serum concentrations and matrices with more physiological mechanical properties being preferable. Furthermore, it is important to consider whether the CAFs being tested are early passage primary cells or have been in culture for several passages and even immortalized. Although certain CAF characteristics are stably maintained in culture, such as their increased ability to remodel the ECM^53^, it is highly likely that some traits are not. Detailed characterization of how CAF properties change on isolation and longer-term culture will help to clarify which functional assays necessitate early passage primary CAFs and which work equally well after longer periods of cell culture.

Matrix production and remodelling can be easily measured. CAFs will produce ECM in culture, and this can be assayed for its composition and quantity using western blotting, quantitative immunofluorescence and mass spectrometry methods^153^. The organization of this matrix can be determined by immunofluorescence, frequently staining for fibronectin, and its mechanical properties can be determined by either atomic force microscopy or shear rheology. Similar techniques can be applied in vivo, with collagen second-harmonic imaging frequently used to assess matrix organization. Multiparametric magnetic resonance imaging (MRI) and magnetic resonance elastography (MRE) can also be used to infer tissue mechanics, with the advantage that these techniques can be translated to clinical imaging and used in clinical trials^154,155^. Histochemical stains to distinguish collagen, including Masson’s trichrome and picrosirius red, provide similar information and the use of crossed polarizing filters during imaging of picrosirius red-stained sections provides a measure of collagen crosslinking^156^. However, most methods for the analysis of pattern lack universal quantitative metrics; in the future, the implementation of methods from network topology analysis and the use of spatial statistics will aid comparison between studies^157,158^.

The secretome of CAFs is typically measured using enzyme-linked immunosorbent assay (ELISA) and cytokine array tools, with a range of standardized commercial reagents available. Exosomes can be analysed following their purification by high-speed centrifugation with clear guidelines on optimal protocols^159^. Crosstalk with cancer cells is usually evaluated in terms of changes in growth and invasion. Cells can be directly co-cultured, with either genetic labels or staining for markers used to distinguish the cancer cells and fibroblasts, indirectly co-cultured (that is, separated by a filter) or conditioned media can be exchanged between separate cultures. Cell number is the most common growth metric, and migration either into a 2D ‘wound’ or across a Transwell are most common invasion metrics. Advances in 3D co-cultures, including the use of organoid cultures and reconstituted matrices, are allowing in vitro assays to more closely mimic the in vivo tissue architecture. In these assays, it should be noted that basement membrane preparations often contain growth factors in addition to matrix components, leading to the possible confounding of matrix and growth factor influences on CAF biology. Pepsinized preparations of collagen I lacking the telopeptide cannot be crosslinked, which leads to altered dependencies on matrix metalloproteinases (MMPs) for cancer invasion^160^. Co-cultures with other cell types from the tumour microenvironment can also be informative. For example, fibroblasts can boost angiogenesis in in vitro assays, with exciting advances in the development of microfluidic angiogenesis models, and an increasing number of studies have shown how they can alter T cell functionality^118,161,162^.

Two main methods are used to explore CAF functions in vivo: transgenic manipulations and co-injection methods. The latter are simpler to perform as they avoid the need for complex mouse crosses. However, there are some notable caveats. The most challenging is that as tumours grow they will contain a mixture of the co-injected CAFs and fibroblasts derived from the host mouse and, for reasons that are not fully understood, host-derived fibroblasts outgrow co-injected CAFs. In practice, this favours the early evaluation of differences between experimental groups and makes it hard to test longer term phenotypes, such as therapy responses. Transgenic manipulations using Cre–*lox* systems to modulate CAFs overcome these issues but have a different set of issues. The most notable of these is the choice of the Cre driver line. Currently, no CAF-specific Cre driver line exists, and even fibroblast-specific Cre driver lines are complex. *Acta2*-Cre and *Acta2*-Cre–ERT can be used, but they will also drive recombination in smooth muscle cells and myoepithelial cells, which poses a particular challenge in mouse models of breast cancer, which has a high frequency of these cell types in the tumour microenvironment. *Fsp1*-Cre has the caveat that fibroblast-specific protein 1 (FSP1; also known as S100A4) is expressed by subsets of myeloid cells. *Pdgfra*-Cre and *Col1a2*-Cre are more generic fibroblast drivers, but the former gene is expressed in some neurons and the latter is expressed in osteoblasts. These issues highlight the importance of using ‘Cre-reporter mice’ to check that recombination is being driven in the intended subset of cells and not more permissively. Following its expression, Cre recombinase can then be used to specifically knock out suitably ‘floxed’ genes in fibroblasts or inhibit or ablate fibroblasts by driving the expression of viral thymidine kinase or diphtheria toxin receptor, respectively.

The other major challenge with in vivo models is how to drive tumorigenesis if the Cre–*lox* system is used to manipulate CAFs. Injection of tumour cells can be used, but this is not always ideal as it bypasses the early stages of tumour initiation. Chemical carcinogenesis is another option, but it is not always easy to control tumour burden, and the cancer genotypes will be variable. Finally, combining Cre–*lox* and Flp–*FRT* (flippase recognition target) recombination systems offers an elegant way to manipulate both tumour and fibroblasts^163^. Once the tumour is established, various metrics relating to CAF function can be measured, including matrix organization and crosslinking, tissue mechanics, tumour vascularization, tumour growth, metastatic spread, immune infiltrate and therapy response. However, this approach is very resource and time intensive, and this poses a barrier for many researchers.

An awareness of the caveats of the assays described above and subsequent improvements to the methods will aid further progress. The use of CAFs that have been established in culture allows molecular perturbations, such as CRISPR gene editing, and the easy repetition of experiments. In the future it will be desirable to determine primary culture conditions that most accurately preserve the in vivo phenotype of CAFs; this is likely to involve considering both the medium and the substrate, with several studies showing how culture in 3D conditions can return fibroblasts to their original phenotype within tissue^10,142^. Combining this with ongoing improvements in the ability to manipulate primary cells will allow assays with human cells that more closely mimic the tumour context. For analysis of interplay with T cells, it will be desirable to isolate cancer cells, CAFs and tumour-infiltrating lymphocytes from the same patient. Improvements in tumour tissue slice culture methods should also be considered for the analysis of CAF biology.

### Reporting CAF metadata

As with all experimental science, the issue of reproducibility is crucial. Research into CAFs is greatly made possible by their ability to be cultured in vitro, but the process of cell culture and the exact conditions can influence cell behaviours. Increased reporting of CAF metadata will improve standardization and robustness in the field. We recommend that studies involving CAFs document the following:

- An absence of the mutations that drive the tumour from which they originate. CAFs may accrue mutations, but it is necessary to exclude that they are simply cancer cells that have undergone EMT. Cancer cells that have undergone a profound EMT clearly warrant detailed study and comparison with CAFs, but these cells should be considered distinct from CAFs.
- The spatial position within the tumour from which the biopsy was taken — central versus margin. If ‘normal’ fibroblasts are isolated at the same time from non-cancerous tissue, then the distance of this tissue from the margin should be recorded.
- Key clinical (stage, grade, prior treatment regimen and driver mutations (if known)) and histological features of the tumour from which the CAFs originate, including ideally staining for CAF markers and the age of the patient or mouse.
- Short tandem repeat profiles of cultured CAFs to allow unambiguous identification of CAFs in subsequent studies. This will mitigate against inadvertent cross-contamination of cultures.
- The passage number of cultured CAFs and the immortalization method used, if any. Details of the culture medium should also be recorded; in particular, serum percentage, addition of exogenous TGFβ, culture substrate or matrix (including type of Matrigel and whether collagen I is telopeptide intact).

## Conclusions

Research into CAFs is at an exciting and critical stage. Accumulating functional analyses in preclinical models and supporting correlative analyses of patient material indicate that improved treatment strategies should be possible by targeting CAFs. Indeed, several clinical trials are under way. However, targeting aspects of the tumour microenvironment has a chequered history, with failures in the area of MMP inhibition, mixed results in targeting angiogenesis and transformative results with inhibition of T cell immune checkpoints in some cancers^164^. Therefore, translating the optimism in the CAF field into real clinical benefits will require careful attention to trial design and tumour sample analysis. Attention needs to be paid to nomenclature and the correct description of different CAF subtypes. This is more than just a semantic issue as the greatest success is likely to come from either targeting specific CAF subsets or interconverting CAF subtypes. Related to this, a better understanding is needed of the relationship between CAFs observed in preclinical models, which often grow very rapidly in young adult mice, as opposed to those in patients, which progress more slowly in an older population. Improved assay standardization and reporting of CAF metadata will assist this endeavour. There are also opportunities to incorporate analysis and reporting of CAF numbers and types in clinical studies that are not primarily focused on fibroblast biology, such as immuno-oncology and targeted therapy trials. This will help to build a more complete picture of the relationship between CAFs and therapy responses and highlight new areas in which combining a CAF-targeted agent with existing therapies could yield greater benefit. With these things in place, we are confident that CAF-targeted therapy will take its place in the toolkit of the oncologist within the next 10 years.

## Glossary

Extracellular matrix: (ECM).The structural network of secreted proteins and glycosaminoglycans that provides structure to tissue.
Angiogenesis: The formation of new blood vessels.
Mesenchyme: A type of tissue composed of loosely associated cells surrounded by extracellular matrix.
Mesoderm: One of three fundamental layers of tissue formed early in development and the predominant source of fibroblastic lineages.
Neural crest: Migratory mesenchymal cells derived from the neural tube and originally the ectoderm.
Adipocytes: Mesenchymal cells specialized for the storage of fat.
Pericytes: Mesenchymal cells that are located adjacent to smaller blood vessels and that support their function.
Focal adhesion kinase: (FAK). A kinase that links integrin extracellular matrix receptors to intracellular changes in cell signalling.
Tumour necrosis factor: (TNF). A cytokine that is produced under conditions of tissue stress and promotes inflammation.
Autophagy: A process of cellular ‘self-eating’ that serves to remove damaged organelles and provide metabolic resources.
Idiopathic pulmonary fibrosis: A life-limiting progressive condition involving persistent activation of fibroblasts and inflammation in the lungs.
Atomic force microscopy: A method for determining the mechanical properties of cells and tissue with high precision.
Shear rheology: The characterization of flow or deformation originating from a simple shear stress field.
Second-harmonic imaging: A label-free method for detection of collagen fibres in both live and fixed tissue.
Magnetic resonance elastography: (MRE). A method for interrogating tissue mechanics in patients.
Short tandem repeat profiles: Variation in repetitive sequences between individuals used to unambiguously identify cell lines.
